# Multi biomarker approach to assess manganese and manganese nanoparticles toxicity in *Pangasianodon hypophthalmus*

**DOI:** 10.1038/s41598-023-35787-0

**Published:** 2023-05-25

**Authors:** Neeraj Kumar, Supriya Tukaram Thorat, Kotha Sammi Reddy

**Affiliations:** ICAR-National Institute of Abiotic Stress Management, Malegaon, Baramati, Pune, 413115 India

**Keywords:** Zoology, Natural hazards, Biomarkers, Risk factors

## Abstract

Manganese (Mn) is an essential element for humans and animals including, fish. It is a still poorly studied in aquatic organisms, where it can be noticeably useful for dietary components and also found pollutant in aquatic environment at high concentrations. On the above information, an experiment was delineated to determine the lethal concentration of manganese (Mn) and manganese nanoparticles (Mn-NPs) alone and with high temperature (34 °C) and its effect on various biochemical markers in *Pangasianodon hypophthalmus*. The median lethal concentration (96-LC_50_) of Mn alone (111.75 mg L^−1^) and along with high temperature (110.76 mg L^−1^), Mn-NPs alone (93.81 mg L^−1^) and with high temperature (34 °C) (92.39 mg L^−1^) was determined in *P. hypophthalmus*. The length and weight of the fish were 6.32 ± 0.23 cm and 7.57 ± 1.35 g. The present investigation used five hundred forty-six fish, including range finding (216 fish) and definitive test (330 fish). The acute definitive doses were applied to assess the effect of oxidative stress, glycolytic biomarkers, protein biomarkers, fish immunity, neurotransmitter, energy level, stress hormone and histopathology. Oxidative stress (catalase, superoxide dismutase, glutathione-s-transferase and glutathione peroxidase), stress biomarkers (lipid peroxidation, cortisol, heat shock protein, and blood glucose), lactate and malate dehydrogenase, alanine and aspartate aminotransferase, a neurotransmitter, glucose-6-phosphate dehydrogenase (G6PDH), ATPase, immune system biomarkers (NBT, total protein, albumin, globulin and A:G ratio) were altered with exposure to Mn and Mn-NPs. The histopathology of the liver and gill were also changed due to exposure to Mn and Mn-NPs. The bioaccumulation of Mn in the liver, gill, kidney, brain and muscle tissues, and experimental water at different intervals of 24, 48, 72 and 96 h were determined. Based on the present results, it is strongly suggested that Mn and Mn-NPs exposure alone and with high temperature (34 °C) enhanced toxicity and altered biochemical and morphological attributes. This study also suggested that essential elements in both forms (inorganic and nano) at higher concentrations of Mn and Mn-NPs lead to pronounced deleterious alteration in cellular and metabolic activities and histopathology of *P. hypophthalmus*.

## Introduction

The population across the world consumes fish in their daily diet due to rich in nutrients such as high-value proteins, vitamins, polyunsaturated omega-3 fatty acids, and minerals. The minerals are essential in small concentrations, but a bit high concentration is toxic in nature^1^. Fish and fisheries product has numerous health benefits. At the same time, it has some contaminants in high concentrations of essential minerals, which pose a severe threat to the aquatic environment and the consumers^2,3^. The minerals are vital for living organisms, including aquatic organisms, required for the physiological role, metabolism, growth, homeostasis, maintenance of acid–base equilibrium, and generation of membrane potential and serve as a cofactor of numerous important enzymes^4^. Manganese (Mn) is an essential trace mineral required to maintain homeostasis such as normal growth, reproduction, vertebral development, and co-factor for many enzymes^5–7^. In contrast, an overdose of Mn produced toxicity to internal homeostasis in living organisms, including aquatic organisms (Fish)^8,9^. This element is also widely used in industries^10^, pesticide formulation^11^, ceramic and glass production, and dry cell^12^. Manganese in higher concentrations could be useful for extracting oil and gases^13^.

All these forms of Mn are discharged into aquatic water bodies and converted into unforeseen toxic metals in aquatic environments^14^. Mn-NPs are used in magnetic resonance imaging and drug delivery in medicine, as ionization-assisting reagents in mass spectroscopy, and most importantly, as biological agents in wastewater treatments and consumer products, such as batteries. The increased production and use of Mn-NPs might increase the potential risk of toxicity through occupational exposure to humans and environment^15,16^. In the present investigation, Mn and Mn-NPs toxicity alone and with high temperature (34 °C) increase the uptake of Mn. Manganese overexposure is more prevalent for induction of toxicity which Mn undergoes an oxi-reduction reaction and may negatively affect the physiology, oxidative stress, and immune systems of the aquatic organism^17^. Similarly, in the case of Mn-NPs, it causes more toxicity than inorganic Mn. It is due to the nature of nanoparticles (NPs) which can stimulate, induce, and multiply in specific cells. Mn in nano-size form is very much smaller than inorganic Mn. However, it can easily pass through the cell membranes, penetrate cellular organelles and interfere with normal cell physiology. These nano-particles characteristics cause damage at the cellular and sub-cellular^18^ and disturb the cell/tissues function. The studies on the toxicity of Mn-NPs in fish are still minimal, and the mechanism leading to toxicity is still unclear. Generally, the metal nanoparticles induce reactive oxygen species (ROS), including superoxide anion, hydroxyl radical (OH^-^), hydrogen peroxide (H_2_O_2_), singlet oxygen (O_2_), and hypochlorous acid as the principle mechanism of cytotoxicity^19^.

Furthermore, considering the prediction of global warming in all ecosystems, including aquatic ecosystems^20^, the temperature affects biological and ecological systems on toxicity in the ecosystem^21^. The elevated temperature has enhanced the toxicity of the nanoparticles, as reported in our previous study^22–24^ in different fish. The fish is a poikilothermic animal; hence, temperature plays a vital role in regulating and maintaining the physio-chemical process in aquatic animal^25,26^. *Pangasianodon hypophthalmus* is the sturdy and best-suitable fish species for toxicity study. It has high market demand and is a good candidate species for diversification of aquaculture and suitable for culture in hard conditions and tolerates high abiotic and biotic stress^23,27^.

At higher concentrations, the Mn and Mn-NPs are toxic and induce oxidative stress (SOD, CAT, GST, and GPx), forming reactive oxygen species (ROS). The Mn and Mn-NPs caused cell injuries, DNA damage and alteration in protein and membranes^28,29^. Its toxicity depends upon availability and concentration^30^. It is protected by scavenging the superoxide radicals and enhancing anti-oxidative status at lower concentrations^31^. The cortisol and blood glucose are essential for maintaining bioenergetics in the organism during stress condition^32^. Neurotransmitters and lipid peroxidation (LPO) are robust biomarkers for aquatic contamination in fish^33,34^. The immunity of the fish is very well concerned in case of stress, viz. total protein, albumin, globulin, and A:G ratio^22,35^. The present investigation was designed to investigate the lethal toxicity of (96 h-LC_50_) manganese (Mn) and manganese nanoparticles (Mn-NPs) alone and along with high temperature (34 °C). Taking into consideration, the present study included the toxic effect of Mn and Mn-NPs on oxidative damage, cellular metabolic stress, histopathology, and bioaccumulation in different fish tissues.

## Material and methods

### Ethics statement

The Research Advisory Committee of ICAR-NIASM approved the study protocol and the endpoints of the experiments. All the methods were carried out by relevant national and international guidelines and regulations and strictly followed the animal research (ARRIVE) guidelines.

### Experimental animal

*Pangasianodon hypophthalmus* was procured from the Biswas hatchery in Kolkata, West Bengal. The fish was transported to the institute in Central Wet laboratory in healthy condition. The length and weight of the fish were 6.32 ± 0.23 cm and 7.57 ± 1.35 g. The potassium permanganate (KMnO_4_) and 1% salt solution were used to quarantine the fish. The fibre-reinforced plastic tanks (500 L) were used to acclimatized the fish for one month and fed with 35% protein diet twice daily. APHA^36^ method was used for analyzing water quality parameters and recorded the normal range for the culture of the fish^35^.

### Toxicity test (median lethal concentration, LC_50_) of Mn and Mn-NPs alone and along with high temperature

The median lethal concentration (LC_50_) of Mn and Mn-NPs alone and with high temperature (34 °C) was determined in *P. hypophthalmus* at 96 h as per the standard method of APHA^37^. The acute test has been static and non-renewable in this bioassay experiment. The manganese acetate synthesized Mn-NPs through a green approach using fish gill. Mn-NPs were used in this experiment to determine the lethal concentration of Mn and Mn-NPs. The range finding test was conducted to determine the acute test. Then a definitive test was performed to determine the median lethal concentration (LC_50_-96 h) of Mn and Mn-NPs with high temperatures. The assay begins with a range finding test with the following concentration viz. 10, 20, 30, 40, 50, 60, 70, 80, 90, 100 and 110 mg L^−1^ with four fish in each replicate and then we have chosen 85, 90, 95, 100, 105 110 and 115 mg L^−1^ with four fish to find out the final range concentration test. The concentration in the range finding test was calculated as 110–115 mg L^−1^ for Mn alone and with high temperature 90–95 mg L^−1^ for Mn-NPs alone and with high temperature. Two hundred sixteen fish were used for the range finding test. Further, 110, 111, 112, 113 and 114 mg L^−1^ concentrations were chosen for Mn alone and with the high-temperature group. Similarly, 91, 92, 93, 94 and 95 mg L^−1^ were chosen for Mn-NPs alone and with the temperature group for definitive test in triplicate with ten fish in each replicate in all experimental groups. Three hundred thirty fish were used for the definitive test. However, five hundred forty-six fish were used in range finding and definitive test. The final concentration of 96 h-LC_50_ was determined for Mn, Mn and temperature exposure group (Mn-T), Mn-NPs and Mn-NPs and temperature exposure group as 111.75, 110.76, 93.81 and 92.39 mg L^−1^ respectively in *P. hypophthalmus* at 96 h. The cumulative mortality was determined based on fish mortality at 24, 48, 72 and 96 h.

### Manganese nanoparticles (Mn-NPs) synthesis using a green approach

#### Preparation of fish tissue extract

The biological method was used for the synthesis of Mn-NPs. The gill tissue was washed in water to remove blood and dust. Then tissues were homogenized (Omni Tissue Master Homogenize, Kennesaw, GA), centrifuged at 5000–6000 rpm to obtain tissue extract, filtered with Whatman paper (0.45 μm pore size) to obtain gill extract^38^.

#### Preparation and characterization of manganese nanoparticles (Mn-NPs)

**Figure 1:**
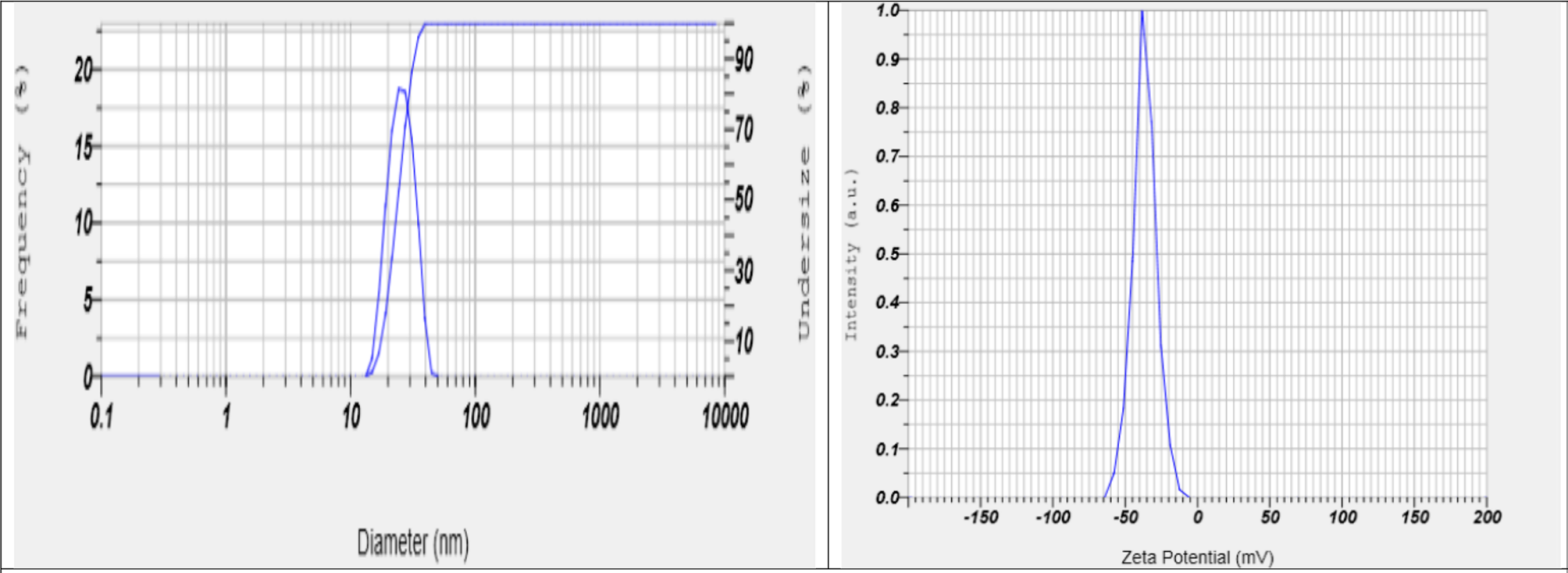
Particle size (25 nm) and zeta potential (− 36.6 mV) of manganese nano particles (Mn-NPs).

**Figure 2:**
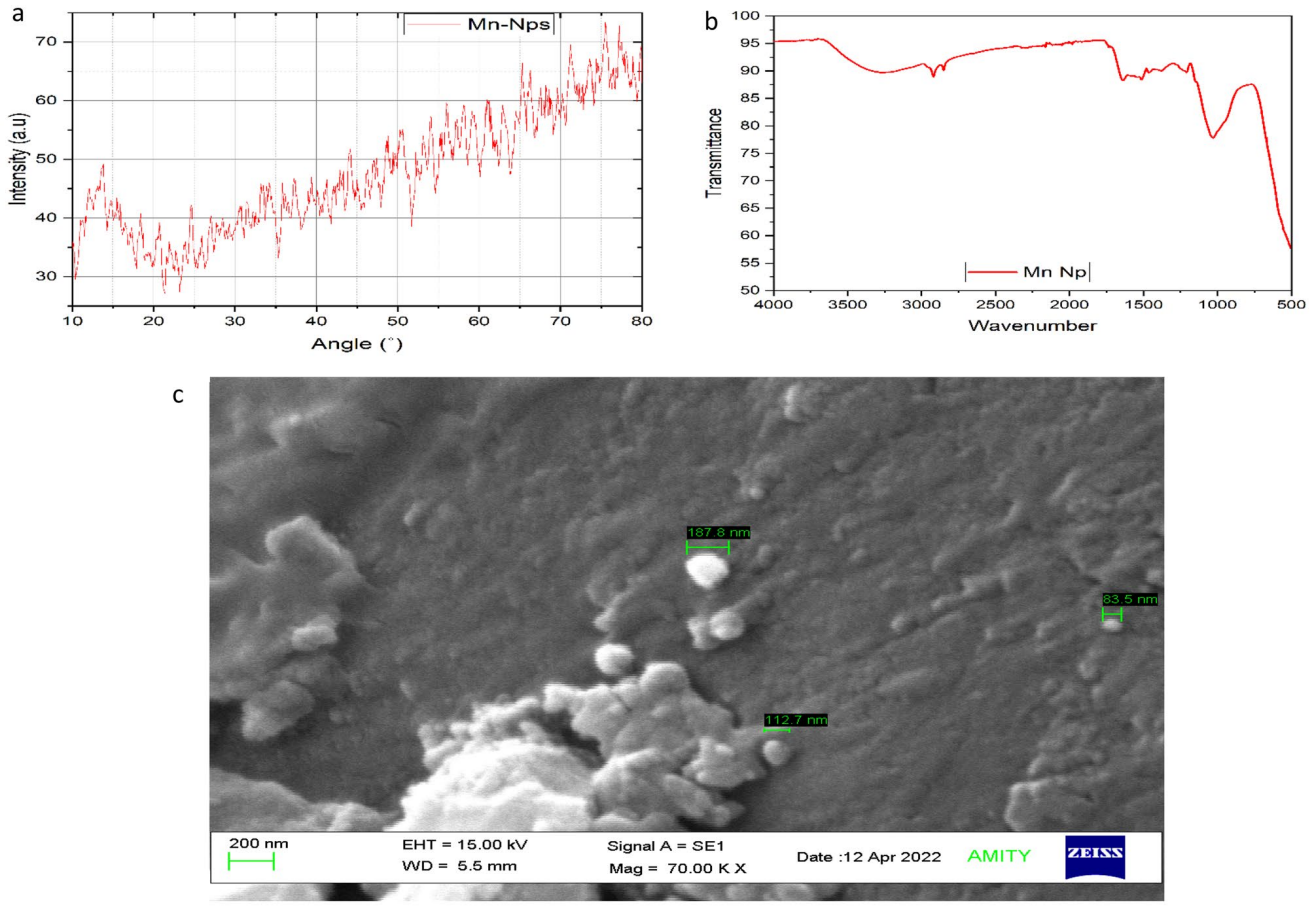
Characterization of Mn-NPs using (**a**) X-ray diffraction (XRD), (**b**) Fourier transform infrared spectroscopy (FTIR) and (**c**) Scanning electron microscope.

The obtained gill extract was mixed with manganese acetate (1 mM) and maintained the solution to pH 6.8. The solution was stirred for 1 h for metal reduction to change the solution to reddish brown, and then mixed curcumin solution (1 mM) for 6 h at room temperature. The colour of the solution changed to permanent black. The permanent black colour indicated the complete stabilization of the manganese nanoparticles. The final solution was washed with de-ionised water to obtain pure manganese nanoparticles and then centrifuged the solution at 5000–6000 rpm. The product was placed in an oven and dried. The characterization was done using a spectrophotometer (Shimadzu, 1900i) at 300–500 nm, and the peak was obtained at 260–380 nm. Then particle was characterized by Nanosize Analyzer (Particle Analyzer, Litesizer 500, Anton Paar, Austria). The mean size and zeta potential were obtained at 25 nm and − 36.6 mV, respectively (Fig. 1). Mn-NPs were also characterized using X-Ray diffraction (XRD), Fourier transforms infrared spectroscopy (FTIR) and scanning electron microscope (Fig. 2).

### Tissues homogenization for enzymatic analysis

Clove oil (100 mg L^−1^) was used for anesthetizing fish during dissection and collected liver, muscle, gill, brain and kidney tissues under aseptic conditions. Then chilled sucrose (0.25 M) (5% w/v) and EDTA 1 mM were used for homogenization (Omni Tissue Master Homogenize, Kennesaw, GA). Then the centrifugation was performed for 20 min at 4 °C at 5000–6000 rpm. Lowry method^39^ was used for tissue protein analysis.

### Oxidative stress

#### Catalase (CAT), superoxide dismutase (SOD) and glutathione-*S*-transferase (GST)

Takahara et al.^40^ was used to determine catalase (EC 1.11.1.6). Briefly, the reaction mixture of 2.45 mL phosphate buffer (50 mM; pH-7), 50 µL tissue homogenate and 1 mL of hydrogen peroxide substrate solution (freshly prepared) was mixed well and the absorbance was read at 240 nm for 3 min. Misra and Fridovich^41^ method was applied to determine superoxide dismutase (EC 1.15.1.1). Briefly, the assay was based on the oxidation of epinephrine- adrenochrome transition by the enzyme. The reaction mixture of 50 µL tissue homogenate, 1.5 mL phosphate buffer and 0.5 mL epinephrine (freshly prepared) was mixed well and immediately read at 480 nm for 3 min in UV spectrophotometer. Glutathione-*S*-transferase (GST; EC 2.5.1.18) activity was measured by the method of Habing et al.^42^ using S-2, 4-dinitrophenyl glutathione (CDNB) as substrate. The method was based on the principle of formation of the CDNB, S-2, 4-dinitrophenyl glutathione adduct and was monitored by measuring the increase in absorbance at 340 nm against blank. Paglia and Valentine^43^ method was used to determine glutathione peroxidase (EC 1.11.1.9). Briefly, 0.2 mL of tissue homogenates were added to 0.2 mL of phosphate buffer, 0.2 mL of EDTA, 0.1 mL of sodium azide and mix well and add 0.1 mL of reduced glutathione and 0.1 mL of hydrogen peroxide. The mixture was incubated in water bath at 37 °C for 10 min. Then centrifuge with 0.5 mL of 10% TCA at 1000 rpm for 5 min and the supernatant was taken out and mixed 2.0 mL buffer and 50 µL DTNB. Then took the reading at 412 nm.

### Lipid peroxidation (LPO)

LPO was determined by the procedure of Uchiyama and Mihara^44^. Briefly, 0.25 mL of homogenate was mixed with 25 μL of 10 mM butylated hydroxytoluene (BHT). 3 mL phosphoric acid (1%) and 1 mL of 0.67% thiobarbituric acid (TBA) were added and the mixture was incubated at 90 °C for 45 min. The absorbance was measured at 535 nm.

### Acetylcholine esterase (AChE, EC. 3.1.1.7)

Acetyl cholinesterase (AChE; E.C 3.1.1.7) was measured by the method of Hestrin^45^. The activity was spectrophotometrically measured as the increase in absorbance of the sample at 412 nm. Acetylcholine chloride and dithiobisnitrobenzoic acid were used as substrate.

### Glycolytic enzymes

#### Lactate and malate dehydrogenase (LDH and MDH)

Lactate dehydrogenase (LDH; l-lactate NAD1 oxidoreductase; E.C.1.1.1.27) was assayed using 0.1 M phosphate buffer (pH 7.5) and 0.2 mM NADH solution in 0.1 M phosphate buffer. The reaction was initiated by adding substrate 0.2 mM sodium pyruvate and absorbance was recorded at 340 nm^46^ (Wroblewski, 1955). A reaction mixture similar to LDH, was used to estimation of malate dehydrogenase (MDH; l-malate: NAD + oxidoreductase: E.C.1.1.1.37) except for the substrate (1 mg oxaloacetate/mL of chilled triple distilled water) and absorbance was recorded at 340 nm^47^.

### Aspartate aminotransaminase (AST) and alanine aminotransaminase (ALT)

ALT (EC.2.6.1.2) and AST (EC.2.6.1.1) activities were measured using the method of Wootton^40^. AST (E.C.2.6.1.1) and ALT (E.C.2.6.1.2) activities were assayed in gill and liver tissue as described by Wooten^48^ (1964). The substrate comprised of 0.2 M d,l-aspartic acid in case of AST and 0.2 M d,l-alanine in case of ALT and 2 mM α-ketoglutarate in 0.05 M phosphate buffer (pH 7.4). In the experimental and control tubes, 0.5 mL of the substrate was added. The reaction was started by adding 0.1 mL of tissue homogenate. The assay mixture was incubated at 37 °C for 60 min. The reaction was terminated by adding 0.5 mL of 1 mM 2,4 dinitrophenyl hydrazine (DNPH) and then 5 mL of 0.4 mL NaOH solution was added, the contents were thoroughly mixed.

### Glucose-6-phosphate dehydrogenase (G6PDH)

G6PDH (E.C.1.1.1.49) activity in gill and liver tissues was assayed by the method of De Moss^49^. The reaction mixture comprised 1.5 mL of 0.1 M Tris buffer (pH 7.8), 0.2 mL of 2.7 mM NADP, 0.1 mL of tissue homogenate, 1.05 mL of distilled water and 0.1 mL of 0.02 M glucose-6-phosphate.

### Total adenosine triphosphatase (ATPase)

Post and Sen^50^ method were used to determine total adenosine triphosphatase (ATPase) (E.C.3.6.1.3). Briefly, the reaction mixture of sodium chloride (0.1 mL), potassium chloride (0.1 mL), magnesium chloride (0.1 mL), ATP (0.5 mL), tissues homogenates (0.1 mL) mixed and incubated for 15 min at 37 °C. Then add TCA (1.0 mL) and centrifuge at 3000 rpm for 5 min and collected supernatant (1 mL), ammonium molybdate (1.0 mL), 2.5 mL distilled water and 0.5 mL of ANSA were mixed. The mixture was incubated for 5 min at room temperature and absorbed the reading at 660 nm.

### Cortisol and HSP-70

Cortisol was quantified in the serum of fish from the different experimental groups through ELISA. The quantification was done using a commercially available Cortisol EIA kit (Catalog no. 500360), procured from Cayman Chemicals, USA. The assay was performed according to the protocol provided along with the kit. The absorbance was read in the ELISA plate reader (Biotek India Pvt. Ltd.). The expression of HSP-70 in gill and liver was determined as per the manufacturer’s instructions (Bioguenix/Enzo Life Science, Mumbai, India). The absorbance was read in the ELISA plate reader (Biotek India Pvt. Ltd.).

### Respiratory burst activity, serum protein and A:G ratio

The respiratory burst activity assay was carried out by Secombes^51^ as modified by Stasiack and Baumann^52^. Total plasma protein was quantified colorimetrically by BCA method. Albumin was quantified using bromocresol green binding method by Doumas et al.^53^. Globulin was quantified by subtracting albumin values from total plasma protein. Albumin/globulin ratio (A/G ratio) was determined by dividing albumin values by globulin values.

### Blood glucose

Blood glucose level was estimated by Nelson^54^ and Somoyogi^55^. Blood was de-proteinized with zinc sulphate and barium hydroxide, filtered and the supernatant is used for glucose estimation. The absorbance was recorded at 540 nm against the blank.

### Manganese analysis from fish tissues and experimental water

Manganese analysis from muscle, gill, liver, kidney and brain were determined using inductively coupled plasma mass spectrometry (ICP-MS) (Agilent 7700 series, Agilent Technologies, USA). The tissues were collected during dissection at the end of 96 h of the experiment. The tissues weighed 0.3–0.5 g for analysis of Mn. The tissues were processed for acidic digestion in HNO_3_ and H_2_O_2_ (5:1) in a microwave digestion system (Microwave Digestion System, Model START-D, SN-135177, Milestone, USA). After that, completely digested, and water samples were filtered with Whatman paper (0.45 µm size) and volume made up to 50 mL to process for Mn analysis through ICP-MS. The multi-element standard solutions of 10 µg mL^−1^ were used to prepare the calibration curve. The calibration curves with R^2^ < 0.999 were accepted for concentration calculation^28^.

### Integrated biomarker response (IBR)

Beliaeff and Burgeot^56^, modified by Sanchez et al.^57^ methods were applied for integrated biomarker response” (IBR). The data were log-transformed (Yi) and then the overall mean (μ) and standard deviation (s) were calculated. Then, Yi values were standardized by the formula: Zi = (Yi − μ)/s, and the difference between Zi and Z0 determined A values. The sum of all the biomarkers calculated the IBR value with respect to all the exposure groups.

### Alkaline single-cell gel electrophoresis (SCGE)/Comet assay

Alkaline single-cell gel electrophoresis and or comet assay was used for DNA damage. The three layers of agarose standardized by Ali et al.^58^ were followed with slight modification. The DNA damage slide was analysed using a fluorescent microscope (Leica Microsystems Ltd, DM 2000, Heerbrugg, Switzerland). The image was analysed using open comet software. The parameter selected for quantification of DNA damage was percent tail DNA (i.e. % tail DNA = 100% head DNA) as determined by the software.

### Histopathology

Roberts^59^ method was used for the histopathology of liver and gill tissues of Mn, and Mn-NPs exposed fish. The histopathological slides were observed under a Microscope (Leica Microsystems Ltd, DM 2000, Heerbrugg, Switzerland).

### Statistics

In the present investigation, the probit analysis was performed using LD50 version 1.1. The data were analysed using IBM SPSS statistics 21 software (SPSS Inc., Chicago, IL, USA). The data were presented as mean ± standard error and analysed through one-way ANOVA with Duncan's multiple range tests for significant difference between the mean with p < 0.05.

###  Consent to publication

Consent was obtained from PME, ICAR-NIASM, Baramati, Pune, Maharashtra, India.

## Results and discussion

### Median lethal concentration

**Figure 3:**
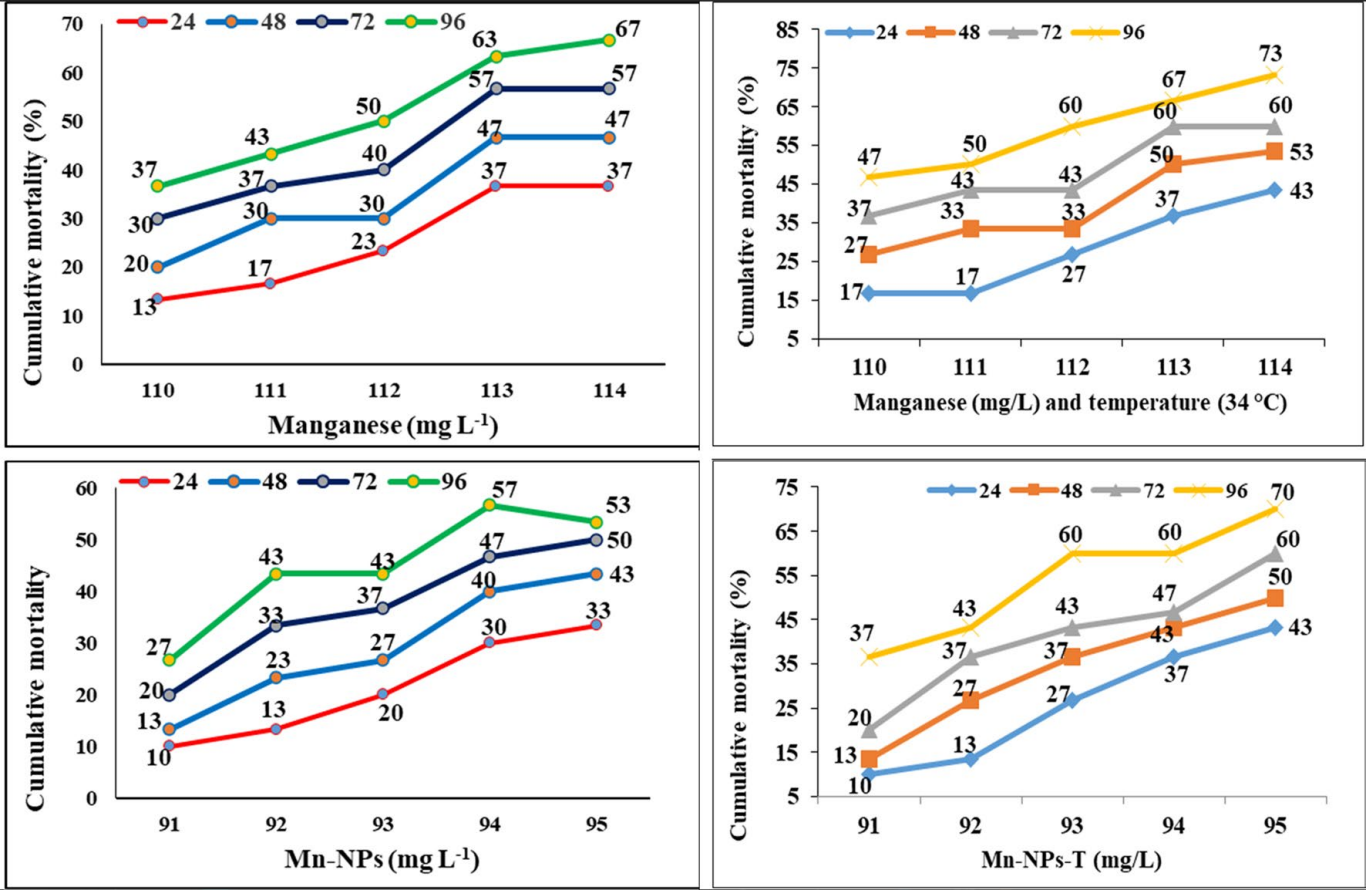
Cumulative mortality (%) of *Pangasianodon hypophthalmus* exposed to different concentrations of Mn and Mn-NPs alone and with high temperature for a period of 96 h (n = 30 for each concentration).

The median lethal toxicity of manganese (Mn) and manganese nanoparticles (Mn-NPs) alone and with high temperature was determined in *P. hypophthalmus*, and cumulative mortality was also calculated at intervals of 24, 48, 72 and 96 h. Data are presented in Table 1 and Fig. 3. The median lethal concentration (96 h-LC_50_) of the Mn exposed group was 111.75 mg L^−1^, and along with high temperature (34 °C) was found to be 110.76 mg L^−1^. Similarly, the 96 h-LC_50_ for Mn-NPs alone and with high temperature was 93.81 and 92.39 mg L^−1^, respectively. The cumulative mortality of the following groups was also calculated at intervals of 24, 48, 72 and 96 h. Manganese treated groups of 110, 111, 112, 113 and 114 mg L^−1^ at 24, 48, 72 and 96 h followed by 13–36, 20–46, 30–56 and 36–66%, respectively and Mn along with high temperature was 16–43, 26–53, 36–60 and 46–73% respectively in concentration and time interval. In the Mn-NPs exposure group of 91, 92, 93, 94 and 95 mg L^−1^, the cumulative mortality was 10–33, 13–43, 20–50 and 26–53% at an interval of 24, 48, 72 and 96 h respectively. Similarly, the Mn-NPs and high-temperature exposure group the cumulative mortality was 10–43, 13–50, 20–60, 36–70% at respective concentration and time interval respectively. Behavioural changes were also observed during the acute toxicity study. The changes were seen as a fast and erratic movement of the fish, the fast opening of the mouth to entrap the oxygen, fast gill movement, loss of body balance, and change in body position, etc^60^.

**Table 1:** Determination of median lethal concentration (LC_50_) of manganese (Mn) and manganese nanoparticles (Mn-NPs) alone and in combination with temperature (34 °C) exposed to *Pangasianodon hypophthalmus* for a period of 96 h.

Exposure (h)	R^2^ value	LC_50_ (mg/L)	95% confidence interval		S-value	Safe level (C)	Intercept	Slope
			Lower	Higher				
Manganese exposure (Mn)								
24	0.67	115.18	113.69	123.67	1.02	34.88	− 72.13	0.67
48	0.69	114.10	112.87	121.15			− 74.94	0.70
72	0.73	112.83	111.72	116.17			− 77.74	0.73
96	0.79	111.75	110.05	112.95			− 84.40	0.80
Manganese (Mn) and high temperature exposure (34 °C)								
24	0.79	114.73	113.43	120.80	1.01	33.33	− 79.33	0.74
48	0.77	113.51	112.37	119.48			− 74.46	0.70
72	0.67	112.21	110.07	115.99			− 66.06	0.63
96	0.82	110.76	105.56	111.88			− 72.53	0.70
Manganese nano-particles (Mn-NP)								
24	0.88	96.64	95.02	105.96	1.03	29.35	− 56.77	0.64
48	0.85	95.47	94.28	100.45			− 68.37	0.77
72	0.77	94.68	93.59	99.80			− 64.47	0.74
96	0.76	93.81	92.55	98.86			− 57.53	0.67
Manganese nano-particles (Mn-NP) and high temperature (34 °C)								
24	0.88	95.36	94.39	98.18	1.01	28.03	− 98.20	0.90
48	0.88	94.71	93.80	97.32			− 97.40	0.90
72	0.91	93.77	92.96	95.28			− 104.0	0.97
96	0.72	92.39	90.77	93.30			− 95.26	0.90

The results showed that Mn-NPs are more toxic than Mn, and the temperature made them more harmful to both the forms of Mn. The Mn toxicity cause impaired motor coordination and brain activity as well as delayed reaction time. The US Environmental Protection Agency has standardized the level of Mn for toxicity range as 0.09 and 0.2 μg m^−3^ and consumed water with Mn contaminated with higher levels of ≥ 10 mg for longer period of time in human^61^. The previous study on determining LC_50_ on different fish species showed a wide range of Mn concentrations in *Oncorhynchus mykiss*. The LC_50_ was 4.8 to 250 mg L^−1^, which depends upon the hardness of the water^62^. In the case of *Heteropneustes fossilis* and *Channa punctata*, the value is 3350 and 3100 mg L^−1^ respectively^63^. The present investigation is the first report to determine the median lethal concentration (LC_50_) of Mn and Mn-NPs alone and with high temperatures (34 °C) in *P. hypophthalmus*. A trace element like manganese is considered essential, but a bit higher concentration makes this element toxic for all organisms, including aquatic organisms. In the present investigation, the Mn and Mn-NPs alone and with high temperature shown behavioural changes in the fish which might be due to altered sensory behavioural and structural damage to olfactory organs as shown in this study^64,65^. Mn-NPs showed more toxicity than Mn, and the along with temperature makes more toxicity. Our earlier study also reported that the temperature enhanced the toxicity of metal^64,65^. In the present study, the toxicity of the Mn and Mn-NPs exposure group depends upon the water's dissolved oxygen availability. The gradient increases in Mn and Mn-NPs, increases the excretion of ammonia in the water, becomes more toxic^66^ and consumes more oxygen during acute stress. In the present investigation, Mn-NPs showed more toxicity than Mn. It might be due to the complex chemical composition compared to Mn. More about the mechanism of toxicity of Mn-NPs is still unclear.

### Oxidative stress and lipid peroxidation (LPO)

The enzymatic activity of oxidative stress enzymes such as catalase, SOD, GST and GPx in liver, gill, kidney and brain and lipid peroxidation (LPO) in liver, gill and kidney of *P. hypophthalmus* exposed to Mn and Mn-NPs are shown in Tables 2–4. Catalase activities in the liver, gill, brain, and kidney were significantly elevated (p < 0.01) in the Mn and Mn-NPs exposed group compared to the unexposed group (Control group). The catalase activities in the liver and gill were substantially higher in Mn-114 mg L^−1^ exposure group and Mn-NPs at 94 and 95 mg L^−1^ groups. In the brain, the catalase activities are significantly higher (p < 0.01) in the exposure group of Mn-113 mg L^−1^ and Mn-NPs-95 mg L^−1^, followed by Mn-114 mg L^−1^ and Mn-NPs-94 mg L^−1^. Further, the catalase activities in the kidney were remarkably higher (p < 0.01) in the exposure group of Mn-114 mg L^−1^, which was higher than Mn-NPs with all exposure groups. Similarly, the SOD activities in the liver and brain were significantly higher (p < 0.01) in the exposure groups of 111–114 mg L^−1^, which was considerably higher than all exposure groups of Mn-NPs (91–95 mg L^−1^). However, SOD activities in the gill and kidney were significantly higher (p < 0.01) in the Mn exposure group compared to the control. Whereas, Mn-NPs exposure group was non-significant (p > 0.05) in the gill and kidney in compared to the control except Mn-NPs-91 mg L^−1^ in gill. Moreover, the GST activities in the liver were significantly higher (p < 0.01) in the exposure group of Mn at 113 and 114 mg L^−1^ and Mn-NPs at 94 and 95 mg L^−1^. Further, the GST activities in the gill, brain and kidney were significantly higher than control and all exposure groups of Mn-NPs. GPx activities in the liver and kidney were noticeably higher (p < 0.01) in the exposure group of Mn at 114 mg L^−1^ compared to control and Mn-NPs exposure groups. Further, the GPx activities in gill were remarkably (p < 0.01) higher in Mn-NPs at 95 mg L^−1^ compared to the Mn exposure group. Similarly, GPx activities in the brain were significantly higher in Mn and Mn-NPs exposure group than in the unexposed group (control group). LPO levels in the liver and kidney were considerably higher (p < 0.01) in Mn treated groups compared to the unexposed (control group) and Mn-NPs-treated groups. However, in the case of gill, the levels of LPO were significantly higher (p < 0.01) in Mn and Mn-NPs treated group compared to unexposed group (control). The level of LPO was significantly similar in Mn and Mn-NPs exposure group.

**Table 2:** Effect of manganese (Mn) and manganese nanoparticles (Mn-NPs) on catalase (CAT) and superoxide dismutase (SOD) in *Pangasianodon hypophthalmus* for a period of 96 h.

Manganese exposure (mg/L)	Catalase (CAT)				Superoxide dismutase (SOD)			
	Liver	Gill	Brain	Kidney	Liver	Gill	Brain	Kidney
Control (Mn-0, Mn-NPs-0)	10.73a ± 1.40	7.20a ± 1.41	10.14a ± 2.39	11.86a ± 1.41	49.26a ± 1.61	35.35a ± 1.05	48.48a ± 1.62	40.30a ± 0.83
Mn-110	22.38c ± 1.03	16.51b ± 1.89	16.96c ± 2.09	17.17b ± 2.13	65.42d ± 2.44	40.19b ± 3.0	71.49e ± 0.69	41.87a ± 1.48
Mn-111	21.42c ± 1.16	20.99c ± 1.23	19.49d ± 1.08	17.98b ± 1.68	69.26e ± 1.96	39.46b ± 0.95	72.01e ± 1.29	43.34ab ± 2.15
Mn-112	27.19d ± 2.64	26.64d ± 1.97	26.04f ± 1.34	22.01c ± 2.42	67.97de ± 2.76	40.15b ± 0.95	69.97e ± 0.81	50.34c ± 1.56
Mn-113	27.57d ± 2.44	29.88de ± 1.25	28.05g ± 1.42	26.21d ± 1.53	67.71de ± 3.55	40.56b ± 1.19	69.21e ± 1.93	46.33b ± 4.04
Mn-114	37.56f ± 2.45	32.77e ± 1.40	26.87f ± 1.71	28.63e ± 2.44	71.38e ± 1.93	42.74b ± 1.04	66.5d ± 1.59	45.36b ± 1.60
Mn-NPs-91	15.38b ± 2.56	15.95b ± 1.71	13.26b ± 2.4	17.29b ± 1.66	70.20de ± 3.64	42.23b ± 2.05	71.49e ± 3.10	44.33ab ± 2.73
Mn-NPs-92	16.88b ± 2.10	21.30c ± 3.08	19.22d ± 2.48	20.09c ± 1.58	56.38b ± 2.09	33.58ab ± 1.26	62.86c ± 1.06	42.03ab ± 1.57
Mn-NPs-93	32.62e ± 2.0	22.25c ± 2.37	23.08e ± 2.50	21.80c ± 2.18	58.37b ± 1.70	35.98ab ± 2.06	57.51b ± 1.27	43.11ab ± 0.65
Mn-NPs-94	37.60f ± 2.6	28.19de ± 2.12	26.98f ± 2.48	23.36 cd ± 2.94	59.24c ± 1.22	36.68ab ± 1.46	58.65b ± 1.42	42.95ab ± 1.05
Mn-NPs-95	35.41ef ± 2.14	29.34de ± 3.03	28.06g ± 1.94	25.36d ± 2.52	58.90c ± 1.40	37.23ab ± 1.74	57.96b ± 1.92	39.81a ± 1.55
p-value	< 0.01	< 0.01	< 0.01	< 0.01	< 0.01	< 0.01	< 0.01	< 0.01

Values in the same column with different superscript (a, b, c, d, e, f, g) differ significantly (p < 0.01). Data expressed as Mean ± SE (n = 6). Catalase and SOD: Units/mg protein.

**Table 3:** Effect of manganese (Mn) and manganese nanoparticles (Mn-NPs on glutathione-s-transferase (GST) and glutathione peroxidase (GPx) in *Pangasianodon hypophthalmus* for a period of 96 h.

Manganese exposure (mg/L)	Glutathione-s-transferase (GST)				Glutathione peroxidase (GPx)			
	Liver	Gill	Brain	Kidney	Liver	Gill	Brain	Kidney
Control (Mn-0, Mn-NPs-0)	0.14a ± 0.01	0.11a ± 0.02	0.14a ± 0.01	0.23a ± 0.02	1.62a ± 0.20	1.13a ± 0.12	0.82a ± 0.06	1.76a ± 0.47
Mn-110	0.25bc ± 0.03	0.22c ± 0.04	0.19b ± 0.03	0.34bc ± 0.06	3.90b ± 0.38	2.94b ± 0.34	2.98b ± 0.38	3.44b ± 0.60
Mn-111	0.29c ± 0.02	0.18bc ± 0.02	0.22bc ± 0.03	0.41c ± 0.02	3.56b ± 0.16	2.97b ± 0.39	3.12b ± 0.18	4.46c ± 0.40
Mn-112	0.39e ± 0.04	0.36de ± 0.02	0.26c ± 0.01	0.40c ± 0.02	4.10c ± 0.60	3.17bc ± 0.41	3.33b ± 0.32	3.59b ± 0.36
Mn-113	0.50f ± 0.05	0.48f ± 0.05	0.35cd ± 0.02	0.30b ± 0.04	4.28c ± 0.38	3.79c ± 0.38	3.48b ± 0.64	4.87c ± 0.45
Mn-114	0.44ef ± 0.05	0.46f ± 0.04	0.38d ± 0.05	0.57e ± 0.04	5.08d ± 0.25	3.85c ± 0.81	4.36c ± 0.16	5.21d ± 0.67
Mn-NPs-91	0.21b ± 0.02	0.16b ± 0.02	0.17ab ± 0.02	0.33bc ± 0.02	3.66b ± 0.33	3.18bc ± 0.52	3.29b ± 0.32	3.99bc ± 0.75
Mn-NPs-92	0.26bc ± 0.04	0.22c ± 0.03	0.23bc ± 0.04	0.39c ± 0.07	3.68b ± 0.51	4.02c ± 0.56	3.68b ± 0.24	4.00bc ± 0.15
Mn-NPs-93	0.34d ± 0.03	0.21c ± 0.04	0.23bc ± 0.03	0.40c ± 0.06	4.52c ± 0.48	3.42bc ± 0.39	3.71b ± 0.31	4.18bc ± 0.43
Mn-NPs-94	0.47f ± 0.02	0.33d ± 0.01	0.26c ± 0.02	0.40c ± 0.04	4.62c ± 0.11	3.37bc ± 0.26	3.77b ± 0.65	4.28bc ± 0.55
Mn-NPs-95	0.39e ± 0.03	0.38e ± 0.02	0.27c ± 0.04	0.48d ± 0.03	4.69c ± 0.21	4.75d ± 0.70	4.07c ± 0.28	4.80c ± 0.46
p-value	< 0.01	< 0.01	< 0.01	< 0.01	< 0.01	< 0.01	< 0.01	< 0.01

Values in the same column with different superscript (a, b, c, d, e, f) differ significantly (p < 0.01). Data expressed as Mean ± SE (n = 6). GST and GPx: Units/mg protein.

Our finding revealed that oxidative stress (catalase, SOD, GST and GPx) in the liver, kidney, and brain and lipid peroxidation in the liver, gill and kidney were altered with acute exposure to Mn and Mn-NPs in *P. hypophthalmus*. The oxidative stress was elevated with exposure to Mn and Mn-NPs might be due to increased production of reactive oxygen species (ROS) such as hydroxyl radical, superoxide radical or hydrogen peroxide^67^. The production of ROS can exceed in the cells resulting in the generation of oxidative stress, which shows various dysfunction due to damage caused by ROS to lipids, proteins, DNA, and alterations in gene expression^68^. In the present investigation, exposure to Mn and Mn-NPs results in overproduction of ROS and dysfunction in mitochondria by blocking the permeability transition pore and reacting with thiol groups (-SH). However, depleting intracellular thiol and creating cellular oxidative stress^69^. Exposure to Mn and Mn-NPs generates oxidative stress, which leads to activating a protective mechanism, which is essential for scavenging produced O_2_^-^ radicals. Thus, elevated production of ROS may be a manifestation of Mn and Mn-NPs toxicity^70^. The enhancement of catalase, SOD, GST, and GPx after exposure to Mn and Mn-NPs might be due to compensation mechanisms arising from metal pollutants^71^. In addition, GST plays a prominent role in the metabolism of aquatic organisms (fish), catalyzing the conjugation of glutathione (GSH) to various electrophiles and functions as a critical defence mechanism against ROS and xenobiotics^72^. The enhancement of oxidative stress enzymes (catalase, SOD, GST and GPx) with exposure to Mn and Mn-NPs reflect the metabolic alteration and damage of the immune system as reflected in our present investigation. GPx is considered the first line of enzymatic defence against Mn and Mn-NPs. Ubiquitous selenium-containing enzymes protect aquatic animals (fish) from oxidative damage. This enzyme not only removes H_2_O_2_ but also reduces lipid hydroperoxides to alcohols against lipid peroxidation^73^. The activation of detoxification systems in the organism has been led by GST, which neutralizes toxic compounds to hydrophilic compounds, thus facilitating their excretion from the organism^74^. The higher activities of oxidative stress enzymes demonstrated that fish try to counteract oxidative effects after exposure to Mn and Mn-NPs. However, the elevated level of lipid peroxidation in fish failed to counteract the oxidative stress entirely, resulting from higher concentrations of Mn and Mn-NPs^75^. Our earlier studies also reported similar findings when the fish were exposed to different inorganic and organic pollutant, the oxidative stress enzymes enhanced significantly^76^. LPO indicates oxidative damage in the cell/tissues from pollutant^77^. The study conducted by Hedayati et al.^75^ reported the same finding with exposure to Mn in *Rutilus caspicus*.

### Cortisol and heat shock protein (HSP-70)

**Figure 4:**
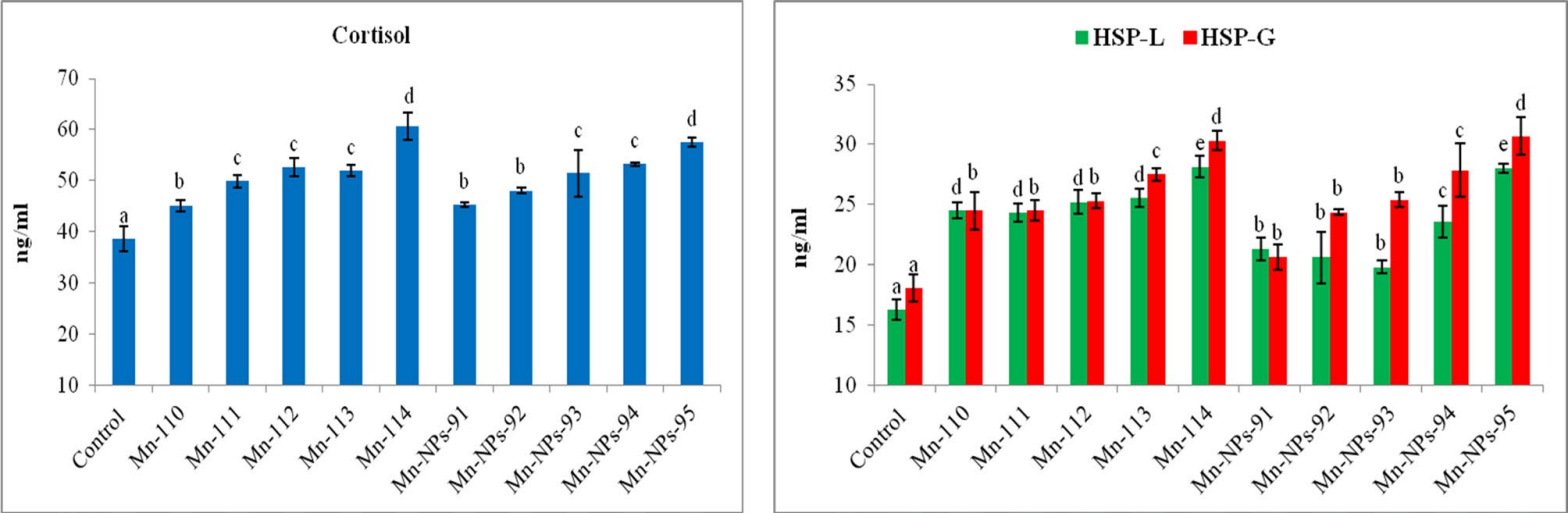
Effect of manganese (Mn) and manganese nanoparticles (Mn-NPs) on cortisol and HSP 70 in liver and gill of *Pangasianodon hypophthalmus* for a period of 96 h. Values in the bar with different superscript (a, b, c, d, e) differ significantly (p < 0.01). Data expressed as Mean ± SE (n = 3).

The levels of cortisol and heat shock protein (HSP-70) of *P. hypophthalmus* exposed to Mn and Mn-NPs during the acute test is shown in Fig. 4. The cortisol level was significantly elevated (p < 0.01) in Mn and Mn-NPs exposure groups in a dose-dependent manner. The cortisol levels were similar in both exposure groups of Mn and Mn-NPs. However, the HSP-70 in the liver and gill showed significant enhancement (p < 0.01) in both Mn and Mn-NPs exposure groups in a dose-dependent manner. The highest levels of HSP 70 was observed in the exposure group of Mn-110 to 113 mg L^−1^ compared to Mn-NPs at 91–94 mg L^−1^.

Exposure to Mn and Mn-NPs elevated the cortisol level could be due to the role of cortisol in glucose regulation via activation of gluconeogenesis and glycogenolysis. Further, the catecholamine released by the chromaffin cell in the fish elevated the glycogenolysis^78^. The cortisol also directly targeted the gill and liver tissue and is essential for mobilising fatty acids, glucose and lipid to maintain homeostasis during stress^79^. While mobilizing the biomolecules, sufficient energy is produced to fulfil the demand of the cell^80^. The cortisol is secreted from the interrenal tissues of fish kidneys and released by the hypothalamus-pituitary-inter-renal axis (HPI axis)^81^. It also stimulates adrenocorticotrophic hormone (ACTH) secretion from the anterior pituitary gland^75^, which helps to release cortisol by inter-renal tissue^72^.

HSP 70 in the liver and gill was elevated with exposure to Mn and Mn-NPs in *P. hypophthalmus*. HSP 70 is the highly conserved ubiquitous protein which suppresses the aggregation of non-native proteins and promotes their refolding and transport to cellular organelles^82^. The elevated HSP 70 in the liver and gill reflects the protective mechanism against stress^83^ as in the present investigation of Mn and Mn-NPs exposure. This also might be due to the induction of oxidative stress in the liver, gill, kidney and brain, damaging these tissues and reducing HSP synthesis resulting in energy deficiency. A similar finding was reported in our previous research on Se and Se-NPs^84^.

### Neurotransmitter enzymes (acetylcholine esterase, AChE)

Acetylcholine esterase (AChE) activities in the brain of *P. hypophthalmus* exposed to Mn and Mn-NPs and presented in Table 4. AChE activities in the brain were noticeably inhibited (p < 0.01) in Mn and Mn-NPs exposed group. AChE activity was significantly inhibited (p < 0.01) from exposure groups of Mn at 110–113 mg L^−1^ and Mn-NPs at 91–95 mg L^−1^.

**Table 4:** Effect of manganese (Mn) and manganese nanoparticles (Mn-NPs) on lipid peroxidation (LPO), acetyl choline esterase (AChE), glucose-6-phosphate dehydrogenase (G6PDH) and ATPase in *Pangasianodon hypophthalmus* for a period of 96 h.

Manganese exposure (mg/L)	Lipid peroxidation (LPO)			Acetyl choline esterase (AChE)	Glucose-6-phosphate dehydrogenase (G6PDH)		ATPase	
	Liver	Gill	Kidney	Brain	Liver	Gill	Liver	Gill
Control (Mn-0, Mn-NPs-0)	6.65a ± 0.53	2.95a ± 0.51	8.84a ± 2.32	0.63e ± 0.01	0.22a ± 0.02	0.27a ± 0.04	109.83a ± 10.37	68.69a ± 8.45
Mn-110	13.00b ± 0.47	8.03b ± 0.59	16.14c ± 1.17	0.43d ± 0.01	0.33b ± 0.05	0.38c ± 0.05	148.79e ± 11.14	91.24c ± 13.35
Mn-111	14.29b ± 0.35	10.54bc ± 0.87	16.63c ± 1.52	0.40d ± 0.03	0.33b ± 0.05	0.39c ± 0.04	150.49e ± 11.63	98.15d ± 9.14
Mn-112	18.85c ± 1.16	12.77c ± 1.0	17.84c ± 1.16	0.39d ± 0.02	0.33b ± 0.01	0.40c ± 0.03	118.97b ± 8.19	106.15e ± 7.94
Mn-113	20.77cd ± 0.48	18.58d ± 0.91	22.51de ± 0.66	0.32b ± 0.01	0.39bc ± 0.03	0.47d ± 0.02	171.96f ± 9.13	95.29cd ± 9.43
Mn-114	24.62d ± 1.13	20.78e ± 1.09	29.85e ± 1.39	0.31ab ± 0.04	0.47c ± 0.05	0.48d ± 0.01	184.67g ± 11.54	143.01h ± 15.38
Mn-NPs-91	11.78b ± 0.73	9.46bc ± 1.07	11.14b ± 1.19	0.41d ± 0.05	0.30b ± 0.03	0.33b ± 0.02	128.53c ± 10.43	78.53b ± 12.61
Mn-NPs-92	12.56b ± 1.10	10.25bc ± 1.16	12.84b ± 0.91	0.42d ± 0.02	0.32b ± 0.07	0.39c ± 0.03	144.53d ± 12.66	97.23d ± 7.39
Mn-NPs-93	13.43bc ± 1.21	12.77c ± 0.67	14.14bc ± 0.54	0.36c ± 0.03	0.37bc ± 0.02	0.43 cd ± 0.04	131.99c ± 8.66	114.19f ± 7.78
Mn-NPs-94	17.80c ± 0.52	18.71d ± 0.44	13.51bc ± 0.32	0.33b ± 0.02	0.44c ± 0.04	0.47d ± 0.08	156.28f ± 9.01	132.13g ± 11.25
Mn-NPs-95	18.29c ± 0.90	19.07de ± 0.92	20.91d ± 1.32	0.27a ± 0.01	0.43c ± 0.03	0.42cd ± 0.06	141.26d ± 10.49	151.52i ± 8.03
p-value	< 0.01	< 0.01	< 0.01	< 0.01	< 0.01	< 0.01	< 0.01	< 0.01

Values in the same column with different (a, b, c, d, e, f, g) differ significantly (p < 0.01). Data expressed as mean ± SE (n = 6). LPO: n mole TBARS formed/h/mg protein, Acetyl choline esterase (AChE): nmole/min/mg protein, G6PDH: Change in 0.01 OD/min/mg protein; ATPase: µg phosphorus released/min/mg protein.

The activity of brain AChE inhibition was determined with exposure to the Mn and Mn-NPs group in a dose-dependent manner, possibly due to the neurotoxic nature of Mn and Mn-NPs. The present investigation's results indicate that AChE is a robust biomarker for Mn and Mn-NPs toxicity^85^. Moreover, the Mn alters the enzymatic systems involved in the cholinergic transmission as AChE and choline acetyltransferase^86^. Acetylcholine esterase (AChE) is the resultant degradative product of Ach and is responsible for the termination of cholinergic response in muscarinic and nicotinic brain ACh receptors^87^. Our previous study also reports the same finding when fish were exposed to Se and Se-NPs to the fish^84^.

### Glucose-6-phosphate dehydrogenase (G6PDH) and adenosine triphosphatase (ATPase)

The activity of G6PDH and ATPase in the liver and gill of *P. hypophthalmus* exposed to Mn and Mn-NPs during the acute test is presented in Table 4. G6PDH activity in the liver and gill was significantly enhanced (p < 0.01) in the Mn and Mn-NPs groups compared to the unexposed group (control). The activities of G6PDH in both the liver and gill were significantly similar in Mn and Mn-NPs exposure groups. The highest activities were observed in the highest concentration in Mn and Mn-NPs exposure group. ATPase activities in the liver were significantly enhanced (p < 0.01) in a dose-dependent manner in the Mn-exposed group except Mn at 112 mg L^−1^, and the activities were higher than in the Mn-NPs exposure. Moreover, in the case of Mn-NPs, the ATPase activities were significantly higher (p < 0.01) in Mn-NPs at 94 mg L^−1^ compared to all other exposure doses. Further, the ATPase activities in gill were significantly higher in (p < 0.01) Mn-NPs at 95 mg L^−1^ exposure group compared to all Mn exposure groups.

G6PDH in the liver and gill was significantly elevated with exposure to Mn and Mn-NPs. It plays a significant role in the pentose phosphate pathway and generates nicotinamide adenine dinucleotide phosphate (NADPH) for antioxidant systems. It is crucial in catalyzing glucose-6-phosphate to 6-phosphoglucose lactone by using NADP as coenzymes and releasing NADPH, essential for the H_2_O_2_ scavenging pathway of cells and glutathione metabolism. In the present investigation, Mn and Mn-NPs might play a role in the induction of G6PDH in response to the pro-oxidant challenge of anti-oxidative status. ATPase is an essential enzyme for energy metabolism, enhancing the Mn and Mn-NPs exposure. The improved demand for ATPase might be due to the inhibiting role of Mn and Mn-NPs in cellular metabolism, mitochondrial respiration and synthesis of adenosine triphosphate (ATP)^88^ and the neurotoxic nature as reflected in AChE results.

### Lactate dehydrogenase (LDH) and Malate dehydrogenase (MDH)

The activities of lactate and malate dehydrogenase in the muscle, liver, gill and kidney of *P. hypophthalmus* exposed to Mn and Mn-NPs during the acute test are presented in Table 5. LDH activities in muscle, liver and gill tissues were significantly higher (p < 0.01) in Mn and Mn-NPs exposure groups compared to the unexposed group (control). In both Mn and Mn-NPs, the activities were significantly elevated in a dose-dependent manner in liver and muscle tissues. Whereas, LDH activities in gill were varies from 129 to 236% in Mn exposure group from 110 to 114 mg L^−1^ and 92–230% in Mn-NPs exposure groups from 91 to 95 mg L^−1^. In case of the kidney, the LDH activities were significantly (p < 0.01) higher in Mn-NPs at 95 mg L^−1^ exposure group compared to the unexposed group and Mn exposure groups. The activities were enhanced in a dose-dependent manner in both exposure groups. In the case of MDH, activities in muscle and liver were significantly higher (p < 0.01) in the Mn exposure group at 114 mg L^−1^ and in the liver at 113 mg L^−1^ compared to the unexposed group and exposure groups of Mn-NPs. In both the organ and exposure group, the MDH activities were significantly elevated (p < 0.01) in a dose-dependent manner except in the liver at 113 mg L^−1^. Further, the MDH activities in the gill and kidney were significantly higher (p < 0.01) in Mn and Mn-NPs exposure group, showing a dose-dependent response.

**Table 5:** Effect of manganese (Mn) and manganese nanoparticles (Mn-NPs) on lactate dehydrogenase (LDH) and malate dehydrogenase (MDH) in *Pangasianodon hypophthalmus* for a period of 96 h.

Manganese exposure (mg/L)	Lactate dehydrogenase (LDH)				Malate dehydrogenase (MDH)			
	Muscle	Liver	Gill	Kidney	Muscle	Liver	Gill	Kidney
Control (Mn-0, Mn-NPs-0)	1.20a ± 0.09	3.07a ± 0.20	1.94a ± 0.28	3.46a ± 0.31	0.17a ± 0.01	0.21a ± 0.01	0.12a ± 0.01	0.14a ± 0.01
Mn-110	3.12c ± 0.22	4.36b ± 0.14	4.44c ± 0.53	4.42b ± 0.79	0.28c ± 0.02	0.29b ± 0.02	0.22b ± 0.02	0.24b ± 0.04
Mn-111	3.43c ± 0.17	4.52b ± 0.23	4.73c ± 0.39	4.44b ± 0.64	0.32c ± 0.04	0.36c ± 0.01	0.29c ± 0.02	0.38 cd ± 0.03
Mn-112	3.96c ± 0.26	4.41b ± 0.32	4.75c ± 0.30	4.73b ± 0.28	0.51e ± 0.03	0.44d ± 0.04	0.39d ± 0.03	0.34c ± 0.03
Mn-113	5.81d ± 0.25	5.25c ± 0.26	4.88c ± 0.44	4.46b ± 0.20	0.44d ± 0.04	0.53e ± 0.06	0.41d ± 0.04	0.46d ± 0.08
Mn-114	6.15d ± 0.27	5.11c ± 0.44	6.52d ± 0.98	5.74c ± 0.27	0.53e ± 0.04	0.49de ± 0.03	0.52e ± 0.04	0.50e ± 0.05
Mn-NPs-91	2.79b ± 0.32	4.02b ± 0.32	3.72b ± 0.23	4.19b ± 0.60	0.23b ± 0.03	0.24ab ± 0.05	0.35 cd ± 0.05	0.31bc ± 0.04
Mn-NPs-92	3.39c ± 0.11	5.03c ± 0.49	4.29c ± 0.61	5.35c ± 0.40	0.19ab ± 0.01	0.29b ± 0.04	0.29c ± 0.04	0.35c ± 0.05
Mn-NPs-93	3.98c ± 0.27	5.57c ± 0.25	3.37b ± 0.30	4.38b ± 0.39	0.33c ± 0.02	0.34bc ± 0.07	0.37 cd ± 0.03	0.39 cd ± 0.04
Mn-NPs-94	5.85d ± 0.26	4.16b ± 0.34	4.82c ± 0.69	5.13c ± 0.32	0.30c ± 0.02	0.36c ± 0.02	0.42d ± 0.02	0.39 cd ± 0.02
Mn-NPs-95	5.57d ± 0.48	5.80c ± 0.10	6.40d ± 0.63	6.88d ± 0.61	0.46d ± 0.01	0.40d ± 0.08	0.48de ± 0.01	0.48de ± 0.02
p-value	< 0.01	< 0.01	< 0.01	< 0.01	< 0.01	< 0.01	< 0.01	< 0.01

Arsenic (As) (mg/L)/ and Temperature (T) (34 °C), Values in the same column with different (a, b, c, d, e, f) differ significantly (P < 0.01). Data expressed as Mean ± SE (n = 6). LDH and MDH: units/min/mg protein at 37 °C.

The target tissues such as muscle, liver, gill and kidney need oxygen for proper functioning and metabolism to surpass the accumulation of several metabolites in the tissues. LDH and MDH were significantly enhanced with exposure to Mn and Mn-NPs could be due to the accumulation of metabolites in different fish tissues in anaerobic conditions. To limit such a situation, it requires energy to maintain its haemostasis^89^; hence, it produces lactate leading to an elevated level of LDH and MDH^90^. Our present investigation shows that Mn and Mn-NPs interact with proteins and enzymes to interfere with an antioxidant defence mechanism, leading to ROS generation and subsequent apoptosis and necrosis^91^.

### Alanine aminotransferase (ALT) and aspartate aminotransferase (AST)

The activities of alanine aminotransferase and aspartate amino transferase in the muscle, liver, gill and kidney of *P. hypophthalmus* exposed to Mn and Mn-NPs are shown in Table 6. ALT and AST activities in muscle and kidney were noticeably higher (p < 0.01) in the Mn exposure groups compared to the unexposed group (control) and Mn-NPs groups, but in the case of the liver, the ALT activities were significantly higher in Mn-NPs exposure group compared to Mn exposure groups and control group. Moreover, ALT activities in gill were substantially higher (p < 0.01) in dose dependent Mn and Mn-NPs exposure groups. Similarly, AST activities in muscle and liver were significantly higher in Mn and Mn-NPs exposure group in dose-dependent way. The activities were significantly similar in both exposure groups of Mn and Mn-NPs. In the gill and kidney, AST activities were substantially higher (p < 0.01) in the Mn exposure group compared to the unexposed and Mn-NPs groups. In both exposure groups, the activities were followed in a dose-dependent manner.

**Table 6:** Effect of manganese (Mn) and manganese nanoparticles (Mn-NPs) on alanine amino transferase (ALT) and aspartate amino transferase (AST) in *Pangasianodon hypophthalmus* for a period of 96 h.

Manganese exposure (mg/L)	Alanine amino transferase (ALT)				Aspartate amino transferase (AST)			
	Muscle	Liver	Gill	Kidney	Muscle	Liver	Gill	Kidney
Control (Mn-0, Mn-NPs-0)	4.60a ± 0.45	12.76a ± 0.58	10.34a ± 0.72	12.10a ± 0.66	6.64a ± 1.02	14.93a ± 0.73	12.40a ± 0.75	11.27a ± 1.95
Mn-110	9.88b ± 1.5	19.70b ± 1.10	15.78bc ± 0.80	19.53c ± 2.07	14.49c ± 1.23	21.26b ± 1.46	18.34b ± 1.06	17.16c ± 1.98
Mn-111	9.57b ± 1.18	23.82c ± 1.27	20.38c ± 1.57	21.62cd ± 0.97	18.04d ± 2.67	24.36c ± 2.06	22.00c ± 2.15	21.15d ± 1.44
Mn-112	13.83c ± 0.37	27.65d ± 1.20	25.39d ± 1.0	29.86e ± 1.22	19.81d ± 2.42	26.87 cd ± 0.82	24.70 cd ± 2.57	22.28d ± 1.94
Mn-113	16.36d ± 1.76	26.65d ± 0.38	25.45d ± 1.16	24.40d ± 2.38	25.11e ± 1.65	26.12 cd ± 2.18	23.05c ± 1.39	24.84e ± 2.27
Mn-114	24.38f ± 1.54	33.62e ± 1.08	23.03cd ± 1.15	32.02f. ± 2.68	24.96e ± 1.60	28.64d ± 0.57	25.47d ± 1.91	24.70e ± 2.48
Mn-NPs-91	8.79b ± 0.74	19.49b ± 0.89	13.92b ± 2.54	16.02b ± 1.25	10.89b ± 0.82	20.92b ± 1.39	17.59b ± 1.32	17.67c ± 1.44
Mn-NPs-92	12.73c ± 1.49	21.04bc ± 3.24	16.37bc ± 2.08	19.74c ± 1.97	13.44c ± 1.44	23.13c ± 1.62	21.69c ± 2.41	14.93b ± 1.72
Mn-NPs-93	16.49d ± 1.85	24.46c ± 1.35	25.19d ± 1.72	18.82bc ± 0.86	19.92d ± 2.58	26.24 cd ± 2.48	24.80 cd ± 2.70	16.86bc ± 3.88
Mn-NPs-94	16.58d ± 1.46	24.84c ± 2.89	23.06cd ± 2.11	20.61c ± 2.49	19.06d ± 2.28	24.01c ± 2.36	19.62b ± 3.40	14.99b ± 1.58
Mn-NPs-95	21.25e ± 3.34	36.12f ± 1.29	24.51d ± 1.37	25.75d ± 2.45	26.33e ± 5.06	25.85cd ± 2.38	21.66c ± 3.66	20.71d ± 2.79
p-value	< 0.01	< 0.01	< 0.01	< 0.01	< 0.01	< 0.01	< 0.01	< 0.01

Values in the same column with different (a, b, c, d, e, f) differ significantly (p < 0.01). Data expressed as Mean ± SE (n = 6). ALT: nmole of sodium pyruvate formed/mg protein/min at 37 °C, AST: nmole Oxaloacetate released/min/mgproteinat37 °C.

AST and ALT activities are potential stress biomarkers indicators for tissue damage^22^. Similar results were reported in the present investigation as liver altered in Mn and Mn-NPs exposed fish. In addition, ALT and AST are linkers between carbohydrate and protein metabolism, essential for using amino acids for oxidation and gluconeogenesis^92^. In the present investigation, the Mn and Mn-NPs could be inhibited many enzymes' activities due to the sulfhydryl (SH) group's binding capability, which is involved in the cellular glucose uptake, gluconeogenesis, fatty acid oxidation, and production of glutathione^93^.

### Immune-related parameters

Immune-related parameters such as NBT, blood glucose, total protein, albumin, globulin and A:G ratio of *P. hypophthalmus* exposed to Mn and Mn-NPs are presented in Table 7. NBT activities were significantly inhibited (p < 0.01) in the exposure groups of Mn and Mn-NPs compared to the unexposed group (control). NBT activities in the Mn exposure groups were significantly lower than in the Mn-NPs exposure group at higher dose of Mn-NPs-95 mg L^−1^. Similarly, the blood glucose was noticeably enhanced (p < 0.01) in Mn and Mn-NPs exposure group. The blood glucose was significantly similar in both exposure groups of Mn and Mn-NPs.

**Table 7:** Effect of manganese (Mn) and manganese nanoparticles (Mn-NPs) on nitroblue tetrazolium (NBT), blood glucose, total protein, albumin, globulin and A:G ratio in *Pangasianodon hypophthalmus* for a period of 96 h.

Manganese exposure (mg/L)	NBT	Blood Glucose	Total protein	Albumin	Globulin	A:G ratio
Control (Mn-0, Mn-NPs-0)	0.70d ± 0.04	74.03a ± 3.40	1.51c ± 0.05	0.35b ± 0.02	1.16d ± 0.03	0.30a ± 0.02
Mn-110	0.54c ± 0.02	84.15bc ± 2.87	1.09b ± 0.01	0.36b ± 0.01	0.73c ± 0.03	0.50c ± 0.04
Mn-111	0.53c ± 0.03	84.66bc ± 0.93	0.86ab ± 0.05	0.32b ± 0.03	0.54ab ± 0.04	0.59d ± 0.03
Mn-112	0.51c ± 0.04	85.15bc ± 2.74	1.02b ± 0.06	0.37b ± 0.04	0.65b ± 0.07	0.58d ± 0.09
Mn-113	0.41b ± 0.02	85.30bc ± 0.65	0.80a ± 0.11	0.22a ± 0.01	0.58ab ± 0.09	0.40b ± 0.05
Mn-114	0.36a ± 0.04	100.46d ± 1.44	0.84a ± 0.05	0.33b ± 0.01	0.50a ± 0.05	0.67e ± 0.07
Mn-NPs-91	0.56c ± 0.02	81.95b ± 2.02	1.02b ± 0.07	0.30b ± 0.02	0.72c ± 0.03	0.42b ± 0.01
Mn-NPs-92	0.54c ± 0.04	81.17b ± 4.10	0.94b ± 0.03	0.30b ± 0.01	0.64b ± 0.05	0.47bc ± 0.02
Mn-NPs-93	0.51c ± 0.03	82.74b ± 1.67	1.03b ± 0.03	0.31b ± 0.03	0.72c ± 0.02	0.43b ± 0.07
Mn-NPs-94	0.52c ± 0.06	88.18c ± 1.86	1.04b ± 0.11	0.31b ± 0.01	0.73c ± 0.06	0.46bc ± 0.09
Mn-NPs-95	0.42b ± 0.04	101.22d ± 2.20	0.72a ± 0.07	0.24a ± 0.03	0.48a ± 0.02	0.52c ± 0.04
p-value	< 0.01	< 0.01	< 0.01	< 0.01	< 0.01	< 0.01

Values in the same column with different (a, b, c, d) differ significantly (p < 0.01). Data expressed as mean ± SE (n = 3). Blood Glucose: mg/dl; NBT: OD at 630 nm.

Further, the total protein, albumin and globulin were significantly reduced (p < 0.01) in Mn and Mn-NPs exposure groups compared to the unexposed group (control). However, levels of these parameters' were significantly similar in the exposure group of Mn and Mn-NPs. The A:G ratio was significantly higher (p < 0.01) in the Mn exposure group than in the unexposed and Mn-NPs groups.

The phagocytic activities (NBT) were reduced with a high concentration of Mn and Mn-NPs due to decreased fish immunity. The total protein, albumin, and globulin were significantly reduced and A:G ratio was enhanced with exposure to Mn and Mn-NPs. The globulin has several heterogeneous proteins, including coagulation factors, transporter proteins, mediators of inflammation and immunoglobulin. The reduced globulin level failed to autologous protective mechanism against autoimmunity through binding to damaged tissues. The albumin, globulin and A:G ratio are indicators of tissue damage, such as liver and kidney disorders, and lower albumin presents the liver damage^94^, supporting our liver histopathology. Blood glucose elevated with exposure to Mn and Mn-NPs indicates the toxic nature of Mn and their nanoparticles and mentions the distress condition. It also showed the stress and nutritional state of the fish^95,96^. The elevated glucose level is also related to the enhanced cortisol level of fish^96^.

### DNA damage in kidney tissues in exposure to Mn and Mn-NPs in fish

DNA damage in the kidney tissue of fish was determined in terms of comet area, comet length, comet DNA, head area, head DNA, head DNA (%), tail area, tail DNA and tail DNA (%) during 96 h of the acute experiment. The data about DNA damage is shown in Table 8. Results of DNA damage reflected that exposure to Mn and Mn-NPs were elevated compared to the control group (unexposed group, Mn and Mn-NPs). In addition, the higher tail DNA (%) was observed in the group exposed to Mn-114 mg L^−1^ followed by Mn-NPs-95 mg L^−1^ compared to the control group.

**Table 8:** Effect of manganese (Mn) and manganese nanoparticles (Mn-NPs) on DNA damage in *Pangasianodon hypophthalmus* for a period of 96 h.

Manganese exposure (mg/L)	Comet area	Comet length	Comet DNA	Head area	Head DNA	Head DNA (%)	Tail area	Tail DNA	Tail DNA (%)
Control	2481 ± 11.45	58 ± 1.69	462,378 ± 25.85	2003 ± 1.63	422,358 ± 94.63	91.34 ± 4.85	478 ± 3.46	40,020 ± 59.45	8.66 ± 0.39
Mn-110	1804 ± 21.32	52 ± 1.05	217,308 ± 14.13	384 ± 11.29	57,130 ± 27.63	26.29 ± 1.08	1420 ± 11.63	160,178 ± 7.63	73.71 ± 2.17
Mn-111	1257 ± 20.06	46 ± 1.63	213,524 ± 11.12	112 ± 1.28	16,331 ± 16.85	7.65 ± 0.85	1145 ± 3.74	197,193 ± 6.45	92.35 ± 3.19
Mn-112	4421 ± 6.18	75 ± 2.74	807,085 ± 1.63	433 ± 5.63	80,520 ± 68.39	9.98 ± 0.39	3988 ± 22.89	726,565 ± 28.63	90.02 ± 1.07
Mn-113	3150 ± 3.56	72 ± 3.41	386,235 ± 2.39	107 ± 1.86	5250 ± 2.74	1.36 ± 0.17	3043 ± 16.59	380,985 ± 31.63	98.64 ± 5.39
Mn-114	495 ± 1.80	31 ± 1.06	26,184 ± 14.32	4 ± 0.21	141 ± 2.85	0.54 ± 0.11	491 ± 3.74	26,043 ± 7.63	99.46 ± 1.46
Mn-NPs-91	670 ± 1.09	31 ± 0.63	57,092 ± 17.32	384 ± 2.95	40,941 ± 6.45	74.84 ± 2.74	317 ± 4.96	21,681 ± 6.11	25.16 ± 1.37
Mn-NPs-92	1513.56 ± 11.63	18.67 ± 1.85	264,490 ± 2.69	522.89 ± 11.63	116,321 ± 25.63	41.97 ± 1.67	1591.78 ± 24.63	252,931 ± 18.63	41.97 ± 2.08
Mn-NPs-93	1594.45 ± 12.71	42.91 ± 2.63	217,279 ± 3.74	514.09 ± 2.85	77,819 ± 18.16	52.66 ± 2.16	1080.36 ± 27.16	139,460 ± 32.63	47.34 ± 1.06
Mn-NPs-94	2236 ± 13.37	52 ± 1.11	394,598 ± 11.36	384 ± 3.48	78,590 ± 51.23	19.92 ± 1.08	1852 ± 2.96	316,008 ± 11.28	80.08 ± 2.17
Mn-NPs-95	820 ± 1.69	32 ± 2.46	65,662 ± 21.52	12 ± 1.08	1735 ± 2.74	2.64 ± 0.62	808 ± 2.96	63,927 ± 7.19	97.36 ± 3.19

Data expressed as mean ± SE (n = 3).

The present study demonstrated that exposure to Mn (110–114 mg L^−1^) and Mn-NPs (91–95 mg L^−1^) induced DNA damage in kidney tissue. Furthermore, Mn and Mn-NPs also induced oxidative stress and apoptosis in the fish. The mechanism behind the induction of DNA damage by Mn is mainly due to its role in neurodegeneration, which is still poorly understood. Still, it is one of the significant factors in inducing DNA damage^97^. It is also claimed that multivalent metallic ions, such as Mn^2+^ and Mn^3+^, readily react with biogenic amines (e.g., dopamine) through Fenton's reactions (redox cycling reactions), thus generating reactive radicals and reactive-oxygen species (ROS) and oxidative damage^98^.

### Bioaccumulation study of Mn in experimental water and fish tissues

The bioaccumulation of Mn in different fish tissues and experimental water at intervals of 24, 48, 72 and 96 h are shown in Table 9. The bioaccumulation of Mn was highest observed in the exposed groups of Mn at 113 mg L^−1^ at 24 h and 114 mg L^−1^ in the 48, 72 and 96 h. Similarly highest concentration was observed in the exposure group of Mn-NPs at 94 mg L^−1^. The bioaccumulation in fish tissues was observed highest in liver and kidney tissues, followed by gill, muscle and brain in both Mn and Mn-NPs exposure groups. The highest concentration was observed in the exposure group of Mn at 112 mg L^−1^ and Mn-NPs at 91 mg L^−1^ in the liver, and gill respectively. Similarly, Mn at 114 and Mn-NPs 93 mg L^−1^ in the case of the kidney. In the brain, Mn at 112 mg L^−1^ and Mn-NPs at 92 mg L^−1^ were higher bioaccumulation. In the case of muscle tissue, the highest concentration was observed in the exposure group of Mn at 114 mg L^−1^ and Mn-NPs at 94 mg L^−1^.

**Table 9:** Determination of manganese (Mn) and manganese nanoparticles (Mn-NPs) concentration in different fish tissues (mg kg^−1^) and experimental water (µL L^−1^) at different time interval.

Manganese exposure (mg L^−1^)	Experimental water (µL L^−1^)				Tissues (mg kg^−1^)				
	24 h	48 h	72 h	96 h	Liver	Gill	Kidney	Brain	Muscle
Control (Mn-0, Mn-NPs-0)	13.54	12.65	12.11	12.78	1.67	2.13	2.78	0.37	0.2
Mn-110	512.23	467.23	448.76	434.61	11.67	9.56	11.67	2.32	4.56
Mn-111	613.45	587.35	548.39	512.76	12.76	8.54	10.98	1.56	5.43
Mn-112	576.65	542.87	527.71	513.89	16.54	10.98	9.78	1.78	5.12
Mn-113	1276.56	1121.72	1134.76	1087.78	13.87	10.45	15.76	1.45	4.13
Mn-114	1217.65	1208.86	1204.61	1196.56	15.65	11.56	16.65	1.65	7.67
Mn-NPs-91	212.32	210.32	208.34	201.34	3.87	2.56	4.43	0.67	2.13
Mn-NPs-92	328.87	332.87	311.67	302.56	2.73	3.72	3.76	0.87	1.78
Mn-NPs-93	207.67	209.82	201.78	198.89	2.95	2.56	4.98	0.42	2.18
Mn-NPs-94	412.37	403.72	398.78	391.42	2.16	4.76	5.64	0.86	2.76
Mn-NPs-95	376.64	372.92	356.73	361.87	2.38	3.29	5.31	0.79	2.96

Data expressed as mean ± SE (n = 3).

In the present study, the water bioconcentration during an acute test at 24, 48, 72 and 96 h in Mn and Mn-NPs exposure group has shown exciting results. The results obtained of the Mn concentration was highest in 113 and 114 mg L^−1^ which is also corroborated with alteration in carbohydrates and protein metabolic enzymes as well as impaired immune systems^74^. The high concentration of Mn changes synaptic processes and the central nervous system due to ions crossing the blood–brain barrier^80^. Similarly, the bioaccumulation of Mn and Mn-NPs in the liver, gill, brain, kidney and muscle were determined. As liver and kidney shown highest bioaccumulation in both the exposure groups. The study conducted by Niemic et al.^99^, the liver and kidney are highly able to bioaccumulate the Mn; hence, it is essential for metabolism as it plays a role in several metabolisms^100^. The vital organ such as gill is direct contact with water; hence, Mn bioaccumulation in this tissue is also directly linked to water absorption, since this organ was constantly contact with the aquatic environment due to the respiratory process^101^. The muscle has the lowest bioaccumulation of Mn because muscle tissues could not have a role in the biotransformation and bioconcentration of metals^102^.

### Integrated biomarker response (IBR)

**Figure 5:**
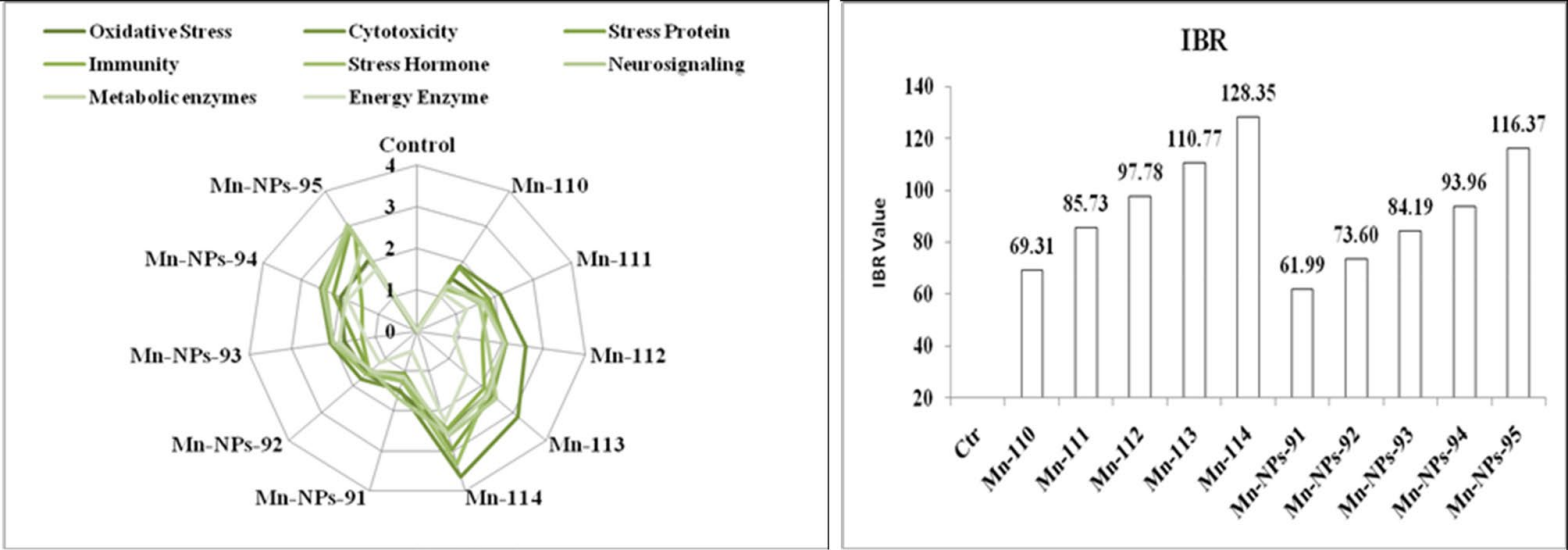
(**A**) Integrated biomarkers response (IBR) of the *P. hypophthalmus* with exposure to Mn and Mn-NPs for a period of 96 h (**B**) Total IBR score.

IBR score for Mn and Mn-NPs treated groups are shown in Fig. 5A,B. IBR score described the effect of all stress biomarkers in this study in different exposure conditions. The highest IBR value was obtained in the group concurrently exposed to Mn-114 mg L^−1^ (IBR, 128.35), followed by Mn-NPs-94 mg L^−1^ (IBR, 116.37). The IBR score for the group exposed to Mn 110 mg L^−1^ to 113 mg L^−1^ was 69.31, 85.73, 97.78 and 110.77, respectively. The IBR score for the Mn-NPs exposure group from 91 to 93 mg L^−1^ was 61.99, 73.60, 84.19 and 93.96, respectively.

The integrated biomarker response (IBR) is the index to summarize the results of a series of different biomarkers scientifically to correlate each and individual to understand and interpret IBR results. This method was developed by Damiens et al.^103^. In the present study, the different biomarkers showed different levels of IBR value, which is dose-dependent. Furthermore, the IBR score increases with other stressors groups of Mn and Mn-NPs compared to the control or unexposed group. Therefore, IBR score could be a helpful tool for quantitative monitoring of pollutant stress levels in fish.

### Depuration and detoxification study of Mn in muscle tissue

**Figure 6:**
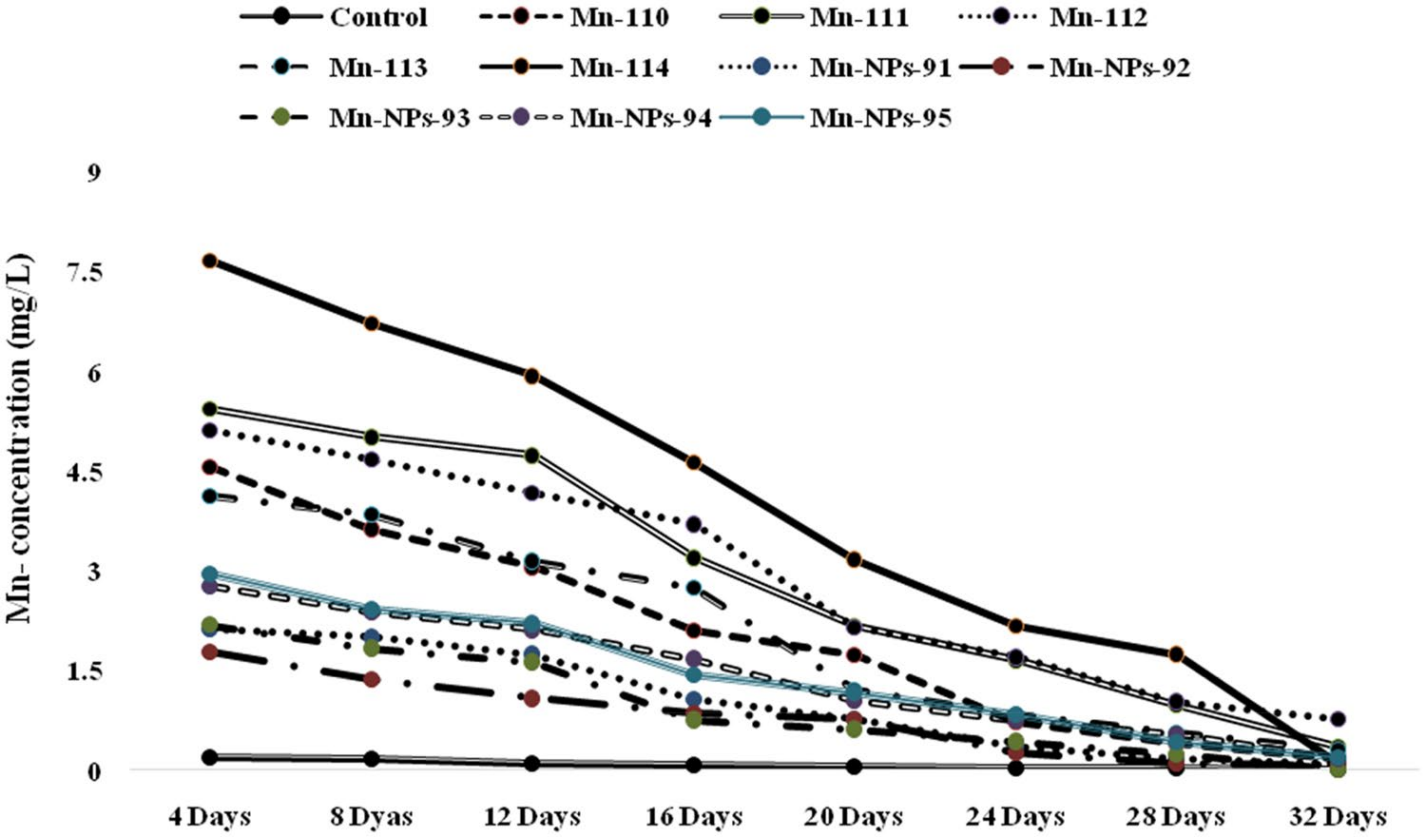
Depuration and detoxification of Mn and Mn-NPs in muscle tissue of *P. hypophthalmus*.

Depuration and detoxification of Mn were performed in the present study, and results are shown in Fig. 6. The depuration and detoxification of Mn started from 8 days (8). The bioaccumulation was occurred upto 8 days, and after that, depuration started in the muscle tissue of the fish. The lowest concentration of Mn was determined during 28–32 days.

In the present investigation, the depuration and detoxification of Mn in muscle tissue took 28–32 days to become normal of in treated groups of Mn-110–114 mg L^−1^. However, it proved that exposure to Mn and Mn-NPs at higher concentrations initiated bioaccumulation in the fish tissues (muscle) and complete depuration in 28–32 days. The depuration of the bioaccumulated metal is the slow process as metal has low efficiency. The depuration also depends upon the number of variables including the fish health status, environmental parameters and nature of the contaminants^104,105^.

### Histopathology

**Figure 7:**
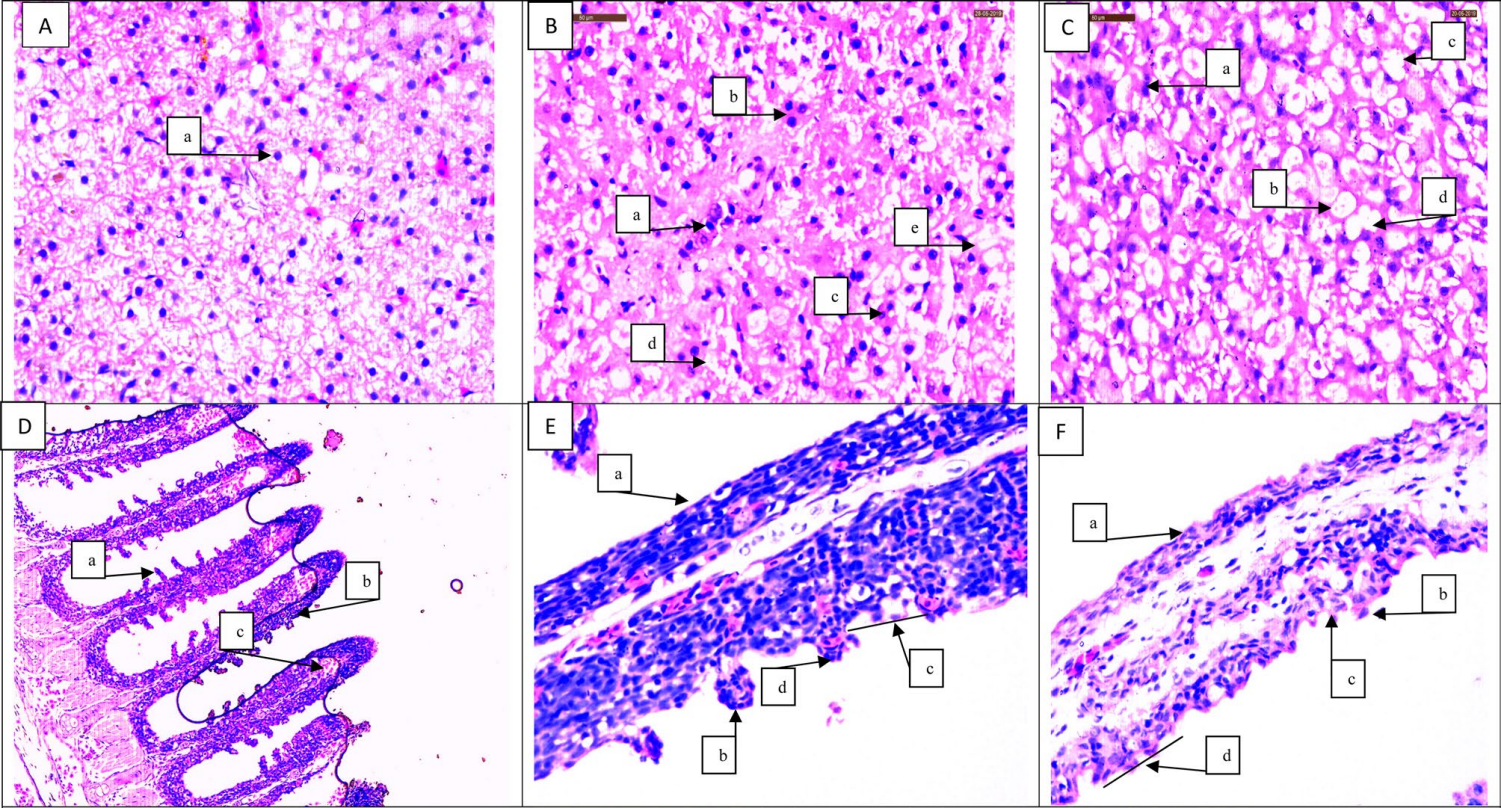
(**A**–**F**) Effect of acute exposure of Mn and Mn-NPs on the histopathology in liver (**A**–**C**) and gill (**D**–**F**) of *P. hypophthalmus* (**A**) (a) Normal architecture of hepatic cell with vesciculated nuclei (**B**) (a) Dilation of sinusoids (b) Degeneration of hepatocyte granular (c) Karyorhexis (d) Necrosis (**C**) (a) pyknotic nuclei (b) multifocal macro vesicular fatty change, steatosis (c) cytoplasmic vacuolization (d) lipid vacuole (**D**) (a) showing normal primary lamellae (b) normal secondary gill lamella (c) pillar cell (**E**) (a) complete destruction of primary gill lamella (b) shortening of secondary gill lamella (c) fusion of primary and secondary gill lamella (d) telangiectasia (**F**) (a) complete destruction of primary gill lamella (b) complete destruction of secondary gill lamella (c) aneurism (d) complete fusion of primary and secondary gill lamella.

The histopathology of the liver and gill of *P. hypophthalmus* exposed to Mn and Mn-NPs during the acute test are shown in Fig. 7A–F. The histopathology of the liver (A-C) has shown several abnormalities such as (A) (a) Normal architecture of hepatic cells with vesiculated nuclei, (B) (a) Dilation of sinusoids (b) Degeneration of hepatocyte granular, (c) Karyorhexis (d) Necrosis (C) (a) pyknotic nuclei (b) multifocal macrovesicular fatty change, steatosis (c) cytoplasmic vacuolization (d) lipid vacuole. Similarly, the structure of the gill (D-F) also has numerous changes due to Mn and Mn-NPs exposure, such as (D) (a) showing regular primary lamellae (b) normal secondary gill lamella, (c) pillar cell (E) (a) destruction of primary gill lamella (b) shortening of secondary gill lamella (c) fusion of primary and secondary gill lamella (d) telangiectasia (F) (a) complete destruction of primary gill lamella (b) complete destruction of secondary gill lamella (c) aneurism (d) complete fusion of primary and secondary gill lamella.

Histopathology is a strong tool to detect the effect of organic and inorganic contaminants in various organs^106^, and treated with chemical contaminants induces several lesions in the liver and gill tissues^107^. The liver is the main organ for the metabolism and detoxification of xenobiotics^108^; hence, it is more chance of degeneration. The fish of the unexposed group showed normal hepatocytes, and Mn and Mn-NPs treated group fish showed abnormalities in both gill and liver tissues. The gill is the primary organ which has regular direct contact with water and is useful for exchanging gases and regulating ionic and acid–base balance and nitrogenous waste excretion^109^.

### Conclusion

The present research finding strongly suggested that a higher concentration of manganese (Mn) and manganese nanoparticles (Mn-NPs) generates severe toxicity during the acute test. Both trace elements (Mn and Mn-NPs) exposure induces toxicity in *P. hypophthalmus* during the median lethal concentration test (96-LC_50_). The effect of Mn and Mn-NPs on biochemical attributes such as oxidative stress, immunity systems, DNA damage, integrated biomarker response and cellular metabolic stress, including histopathology and bioaccumulation and depuration of Mn and Mn-NPs on different tissues level, which shown biochemical markers for metal contamination. The present investigation is the first to report toxicity of Mn and Mn-NPs alone and with high temperature in *P. hypophthalmus* and a specific biomarker for detecting Mn and Mn-NPs. Further, Mn is an essential micronutrient for humans and animals, including fish. Hence, this study recommended Mn should be used in less concentration and nanotechnology (Mn-NPs) can help in the most effective way to minimize Mn levels with high bioavailability in fish.

## Data Availability

The datasets generated during and/or analysed during the current study are available from the corresponding author on reasonable request.
